# E-Learning to Improve Suicide Prevention Practice Skills Among Undergraduate Psychology Students: Randomized Controlled Trial

**DOI:** 10.2196/14623

**Published:** 2020-01-22

**Authors:** Marie-Louise J Kullberg, Joanne Mouthaan, Maartje Schoorl, Derek de Beurs, Robin Maria Francisca Kenter, Ad JFM Kerkhof

**Affiliations:** 1 Department of Clinical Psychology Leiden University Leiden Netherlands; 2 Netherlands Institute for Health Services Research Utrecht Netherlands; 3 Department of Clinical Psychology, Faculty of Psychology University of Bergen Bergen Norway; 4 Vrije University Amsterdam Amsterdam Netherlands

**Keywords:** e-learning, suicide prevention, digital learning, skills training, randomized controlled trial, undergraduate students

## Abstract

**Background:**

Despite increasing evidence of the effectiveness of digital learning solutions in higher vocational education, including the training of allied health professionals, the impact of Web-based training on the development of practical skills in psychiatry and psychology, in general, and in suicide prevention, specifically, remains largely understudied.

**Objective:**

This study aimed to determine the effectiveness of an electronic learning (e-learning) module on the adherence to suicide prevention guidelines, knowledge of practical skills, and provider’s confidence to have a conversation about suicidal behavior with undergraduate psychology students.

**Methods:**

The e-learning module, comprising video recordings of therapist-patient interactions, was designed with the aim of transferring knowledge about suicide prevention guideline recommendations. The program’s effects on guideline adherence, self-evaluated knowledge, and provider’s confidence were assessed using online questionnaires before the program (baseline and at 1 month [T1] and 3 months after baseline). The eligible third- and fourth-year undergraduate psychology students were randomly allocated to the e-learning (n=211) or to a waitlist control condition (n=187), with access to the intervention after T1.

**Results:**

Overall, the students evaluated e-learning in a fairly positive manner. The intention-to-treat analysis showed that the students in the intervention condition (n=211) reported higher levels of self-evaluated knowledge, provider’s confidence, and guideline adherence than those in the waitlist control condition (n=187) after receiving the e-learning module (all *P* values<.001). When comparing the scores at the 1- and 3-month follow-up, after both groups had received access to the e-learning module, the completers-only analysis showed that the levels of knowledge, guideline adherence, and confidence remained constant (all *P* values>.05) within the intervention group, whereas a significant improvement was observed in the waitlist control group (all *P* values<.05).

**Conclusions:**

An e-learning intervention on suicide prevention could be an effective first step toward improved knowledge of clinical skills. The learning outcomes of a stand-alone module were found to be similar to those of a training that combined e-learning with a face-to-face training, with the advantages of flexibility and low costs.

## Introduction

### Background

Suicide is currently one of the most common causes of death in the Netherlands, with rates of approximately 11 cases per 100,000 inhabitants since 2013 [1,2]. With suicide being an obvious public health concern, health care professionals need to direct efforts toward reducing suicide rates. However, health professionals are not well equipped because they receive little training that specifically prepares them to address suicidal behavior. For example, a common misperception prevails that asking about suicidality could evoke suicidal thoughts [3] even though studies have shown that asking patients about suicidality can evoke feelings of relief and openness, which in turn can ensure timely treatment and prevent exacerbation of symptoms [4,5]. Therefore, the clinician’s proficiency in discussing suicidal thoughts is crucial for estimating the risk for suicide attempts, and so is the clinician’s confidence in exercising these specific skills.

The advances in technology, rising costs in health care, and need for continuous education of allied health professionals have made electronic learning (e-learning) a popular educational method, specifically in skills education [6-8]. In both undergraduate and graduate medical programs, the use of e-learning modules is widespread [9-11]. It is used successfully in several branches of medicine and specialties, such as dermatology and surgery [12], as these modules allow for the teaching of concrete and measurable skills such as skin examination. In comparison, e-learning has been applied less frequently in the field of psychiatry [9,11] because direct patient contact is deemed most instructive to master interpersonal professional skills [13]. However, the results of a systematic review on the effectiveness of e-learning programs on clinician behavior and patient outcomes showed that e-learning programs are as effective as traditional learning approaches [14] and show superior results in improving health care professional behavior of nurses and nursing students when compared with no instruction [15]. More recently, the results of a pilot randomized controlled trial in medical students showed that an e-learning course was successful in enhancing mental health first-aid intentions in medical students, suggesting the potential of the e-learning course to improve mental health first-aid skills [16]. Furthermore, positive results in cost-effectiveness have made e-learning a more popular tool for therapists in mental health [17]. The University of Oxford has developed a Web-centered training for enhanced cognitive behavioral therapy for eating disorders, which successfully trained a large number of therapists [18]; for example, of the 102 therapists who commenced the training program, more than 80% completed the training, and their scores increased substantially on the validated measure of competence [17].

### This Study

Suicide is known to be a complex issue that requires a range of prevention initiatives and methods of evaluation [19]. In the Netherlands, mental health institutions are joining forces to make e-learning for mental health care professionals (in training) on therapeutic skills widely available [20]. As part of a proven effective e-learning–supported Train-the-Trainer intervention named Professionals in Training to STOP (PITSTOP) suicide, an e-learning module was developed to appropriately discuss suicidality in a professional context and offered in conjunction with face-to-face training [21-23]. Considering the positive results of PITSTOP suicide on professionals and the benefits of e-learning in mental health education [24,25], this study aimed to determine the effectiveness of the singular use of the e-learning module on knowledge of suicide prevention, provider’s confidence, and adherence to the Dutch guideline on the assessment of suicidal behavior [26] in undergraduate psychology students.

## Methods

### Design and Procedure

A randomized controlled trial was conducted among the third- and fourth-year undergraduate clinical (neuro-) psychology students of the Vrije Universiteit (VU) University in Amsterdam, The Netherlands, who were fluent in Dutch. Students at that particular stage in their curriculum were deemed to be eligible to follow an e-learning module on suicidal behavior because they would be preparing for their internship and their professional career as a health care professional. They were recruited via the student administration office. All participants were informed about the study and provided online informed consent. The study was approved by the ethical committee of the Department of Clinical Psychology at the VU University.

Baseline (T0) questionnaires were sent to each participant. Subsequently, participants were randomized to either the intervention or the waitlist control condition using the randomize subset option in Qualtrics. In the intervention condition, participants received both a message in Qualtrics and an email with the link and the log-in code for the e-learning module. Follow-up questionnaires were sent 1 and 3 months after the baseline questionnaires (T1 and T2). The participants in the waitlist control condition received a message via Qualtrics, thanking them for the completion of the first questionnaire (T0) and informing them about a follow-up questionnaire after 1 month. After completing the T1 follow-up, participants in the waitlist control group also received access to the module, with a final follow-up at T2. All participants were blind to their condition. As an incentive to finish the module, all participants could request a certificate when they finished at least two assessments and had followed the e-learning module for at least half an hour.

### Intervention

The e-learning module was originally developed to support a face-to-face training called PITSTOP suicide [21]. The content of both the face-to-face training and the e-learning module was based on the Dutch multidisciplinary guideline on the assessment and treatment of suicidal behavior [26]. The core of the module consisted of 5 videos in which experienced nurses, psychologists, and psychiatrists interacted with different patients with suicidal tendencies, played by actors, to teach the guideline recommendations. The patients were of various ages, gender, and diagnostic categories and displayed various prototypical suicidal symptoms, cognitions, and interaction problems. In designing the video content, the dialogues were purposely unscripted to enable more realistic scenes and to prevent the professionals from acting. In addition, the videos included vignettes, guideline topics, and recommendations that were explained by experts on the topic. The psychiatrists, psychologists, and nurses were selected from the clinical staff of departments participating in the PITSTOP study to act as a role model in the module. During the PITSTOP study, trainees had personalized access to the e-learning module that could be viewed repeatedly. The total running time of the module was 60 min.

### Outcome Measures

To assess *guideline adherence*, students rated 5 video vignettes of 30 seconds, in which experienced nurses, psychologists, and psychiatrists interact with suicidal characters, played by actors, on the likelihood that they would respond with any of the 25 different interventions on a visual analog scale (ranging from 1 to 100, with 1=very unlikely and 100=very likely). The examples of interventions are “Ask whether the patient thinks about suicide” and “Ask how hopeless the patient is feeling.” At T0, T1, and T2, participants rated the same vignettes. The mean item score of the 125 items (25 items × 5 vignettes) was used as an estimate for guideline adherence, which ranged from 0 to 100, with higher scores representing stronger guideline adherence. The internal consistency of the guideline adherence scale appeared to be excellent (Cronbach alpha=.96) in this sample.

The *knowledge of suicidal behavior* was measured by the 7-item subscale self-evaluation of knowledge on suicidal behavior of the 14-item Question-Persuade-Refer questionnaire [27]. The students were asked to rate the extent to which their knowledge increased on items including “Suicide warning signs,” “How to ask someone who may be suicidal,” and “Persuading someone to get help” on a 5-point scale from 1 (very little) to 5 (a lot). Cronbach alpha was .86, reflecting a good internal consistency.

The *provider’s confidence* was calculated by summing the scores of the 2 items “I am confident in my ability to successfully assess suicidal patients” and “I am confident in my ability to successfully treat suicidal patients” [28]. The response options were strongly disagree, disagree, neutral, agree, and strongly agree (ranging from 1 to 5).

For the *evaluation of the e-learning module*, participants were asked regarding the extent to which they agreed with the following statements (range 1-10), rating from totally disagree to totally agree: “I learned a lot about prevention of suicide,” “The module is a good preparation for my internship/future/job as psychologist,” “I would recommend the module to my colleagues and fellow students,” “I feel confident dealing with people with suicidal thoughts,” and “I appreciate the e-learning module.” The mean score of the 5 items was used to express the evaluation of the e-learning.

### Sample Size

To calculate the sample size, we used the effect size (Cohen *d*) of data from the PITSTOP suicide study on provider’s confidence. The effect size in PITSTOP suicide study was 0.5. To be able to detect this effect size, assuming an alpha of .05 and the statistical power of 1−beta=.80, we set the total number of participants to be included to 128. To allow for attrition, more than 128 participants were included.

### Analysis

Baseline characteristics were compared between groups using independent sample *t* tests and chi-square tests. A missing values analysis was conducted to assess the patterns of missing values. Data were imputed using multiple imputation with 10 imputations and 100 iterations, using the *mice*-package [29]. To assess the effect of the module on the levels of guideline adherence, self-evaluation of knowledge, and provider’s confidence, we performed linear regression analyses with scores at T1 as outcome, scores at baseline as covariate, and condition as between-subjects variable.

To assess the follow-up effect of the module (ie, when both conditions had received the module), we performed paired sample *t* tests between T1 and T2 separately for each condition on the completers-only data as a sensitivity analysis. All analyses were performed with SPSS Statistics 25.0 (IBM Corp, Armonk, New York, 2019) and R version 3.5.0 (R Core Team, Vienna, Austria, 2018).

## Results

### Sample Characteristics

T0 were filled in by 398 participants, from which 211 were randomized to the intervention group and 187 to the waitlist control group. Participants in the intervention group reported higher levels of self-evaluated knowledge (*t*_396_=2.1; *P*=.03), and in the waitlist control group, more participants had already completed their practical clinical internship (χ²_1,398_=4.5; *P*=.04). No difference regarding other baseline characteristics was noted in the groups (see Table 1).

**Table 1:** Group differences at baseline and at 1 month and 3 months after baseline on characteristics and outcome measures (N=398).

Characteristics	Intervention group (n=211)	Waitlist control group (n=187)	*P* value
Female gender, n (%)	180 (85.3)	156 (83.4)	.68
Age (years), mean (SD)	22.1 (4.0)	22.1 (3.0)	.99
Completed internship, yes, n (%)	58 (27.5)	70 (37.4)	.04^a^
T0^b^ Guideline adherence, mean (SD)	57.4 (11.5)	58.9 (11.4)	.19
T1^c^ Guideline adherence, mean (SD)	62.1 (15.6)	59.8 (14.7)	<.001^a^
T2^d^ Guideline adherence, mean (SD)	62.0 (13.4)	63.7 (10.0)	.47
T0 Self-evaluation of knowledge, mean (SD)	17.3 (4.6)	16.3 (4.6)	.03^a^
T1 Self-evaluation of knowledge, mean (SD)	22.3 (4.6)	16.4 (5.5)	<.001^a^
T2 Self-evaluation of knowledge, mean (SD)	22.9 (3.6)	22.1 (3.9)	.26
T0 Provider’s confidence, mean (SD)	5.1 (1.8)	5.0 (1.7)	.61
T1 Provider’s confidence, mean (SD)	6.5 (1.7)	4.6 (1.9)	<.001^a^
T2 Provider’s confidence, mean (SD)	6.5 (1.5)	6.0 (1.7)	.23

^a^*P* values<.05 were considered significant.

^b^T0: baseline.

^c^T1: 1 month after baseline.

^d^T2: 3 months after baseline.

### Dropout and Missing Data

**Figure 1:**
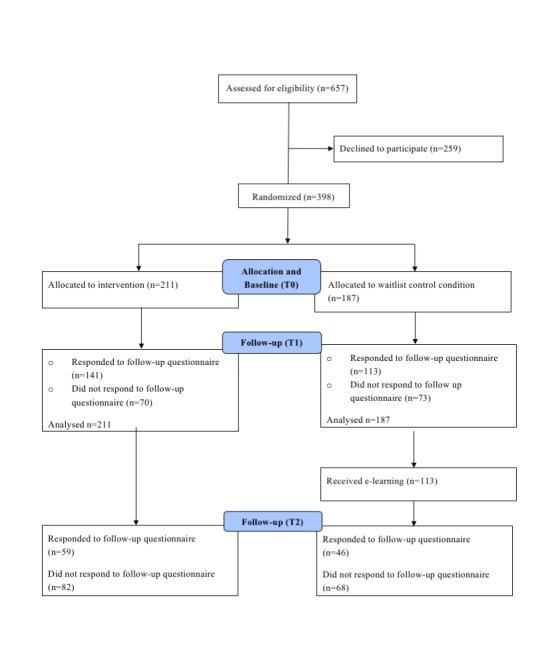
Flow of students through the trial. T0: baseline; T1: 1 month after baseline; T2: 3 months after baseline.

Figure 1 shows the flow of students through the trial. Of the 211 intervention group participants, 141 (66.8%) completed the T1 follow-up, and 59 (28%) completed the T2 follow-up. In the waitlist control group (n=187), 113 participants (60.5%) completed the T1 follow-up. After T1, the participants in the waitlist control condition were offered the intervention, of which 46 (25%) used the e-learning and completed the T2 follow-up.

Dropout rates at T1 in the intervention condition were higher than those in the waitlist control condition and were comparable in both conditions at T2. Values at T1 and T2 were not missing at random according to the Little’s missing completely at random test (χ^2^_11_=22.8; *P*=.02). Baseline characteristics were used to predict missing values at follow-up. Completers were younger than noncompleters at T1 (*t*_180_=–2.4; *P*=.02) and T2 (*t*_330_=–2.2; *P*=.026). Completers showed higher levels of provider’s confidence than noncompleters at T1 (*t*_301_=2.2; *P*=.03). For all other baseline characteristics, noncompleters did not differ from completers (all *P* values>.05).

### Imputed Data: Effect of Module on Guideline Adherence, Confidence, and Knowledge

After receiving the e-module (T1), participants in the intervention group reported higher levels of self-evaluated knowledge (beta=5.3; SE=0.43; *d=*1.6), guideline adherence (beta=3.5; SE=1.0; *d*=0.15), and provider’s confidence (beta=1.7; SE=0.17; *d=*1.1) than those in the waitlist control group. Baseline levels of knowledge, confidence, and guideline adherence were included as covariates in the regression analyses.

### Completer Analysis

When completers-only data (intervention group: n=141 and waitlist control group: n=113) was used at T1, the intervention group scored higher on guideline adherence (beta=3.5; 95% CI 1.7-5.8; *d*=0.5), knowledge (beta=5.3; 95% CI 4.5-6.1; *d*=1.4), and confidence (beta=1.7; 95% CI 1.3-2.0; *d=*1.1) than the waitlist control group. Provider’s confidence in dealing with suicidal behavior (*t*_140_=9.9; *P*<.001), guideline adherence (*t*_140_=6.19; *P*<.001), and self-reported knowledge (*t*_140_=15.42; *P*<.001) significantly increased in the participants in the intervention group after completion of the e-learning module.

Comparison of the scores at T1 and T2 follow-up that were made using completers-only data showed that levels of knowledge, guideline adherence, and confidence within the intervention group stayed constant (all *P* values>.05), and the levels of the outcome variables, such as knowledge (*t*_45_=7.6; *P*<.001), guideline adherence (*t*_45_=2.5; *P*<.018), and provider’s confidence (*t*_45_=2.5; *P*=.002), within the waitlist control group improved significantly after receiving the e-learning module.

### Evaluation of the Module by Completers

Participants who completed the intervention (N=102) filled in the evaluation questionnaires at T2, indicating the extent to which they agreed with the statements evaluating the e-learning module. The results show evaluation scores ranging from 6.2 for the statement “I feel confident dealing with people with suicidal thoughts” (SD 1.9) to 7.5 for the statement “I appreciate the e-learning module” (SD 1.5). In addition, the participants rated the other statements in a moderately positive manner, for example, “I learned a lot about prevention of suicide” (mean 6.8, SD 1.4), “The module is a good preparation for my internship/future/job as psychologist” (mean 6.9, SD 1.4), and “I would recommend the module to my colleagues and fellow students” (mean 7.1, SD 1.5).

## Discussion

### Principal Findings

This study aimed to test the effectiveness of a suicide e-learning module in undergraduate students with respect to the knowledge of suicide prevention, provider’s confidence to have a dialogue about suicide, and adherence to the national guidelines on the assessment of suicidal behavior [26]. One month after completing the e-learning module, the students in the intervention condition reported feeling more confident and knowledgeable compared with those in the waitlist control group who did not have access to the e-learning module. Completers in the intervention condition demonstrated better guideline adherence, knowledge, and provider’s confidence than waitlist controls. The learning effects of the e-learning module were maintained at the 3-month follow-up. These results were replicated in participants in the waitlist control condition who completed the e-learning module after T1. The results were comparable with the improvement in provider’s confidence, guideline adherence, and self-reported knowledge in mental health care professionals who received both e-learning and face-to-face training [22,24]. These findings are surprising as well as encouraging because we expected smaller effect sizes in students compared with professionals owing to the intense 1-day face-to-face training that accompanied the e-learning for the professionals [23]. Owing to less clinical experience, the baseline skills and knowledge level of students were lower than that of the professionals and may have provided more room for improvement. Hence, the effects of additional face-to-face training in this group of undergraduate students above those of the e-learning module are worth investigating.

Furthermore, the results were also comparable with those of a study evaluating an e-learning module on suicide prevention in mental health providers in the United States [30]. In this study, the effects of e-learning were similar to that of an in-person training on suicide prevention. The effect sizes of guideline adherence were considered small; however, in this study, approximately 10% change in guideline adherence, provider’s confidence, and knowledge was found, which is consistent with other studies in general medicine and psychiatry on the effect of educative interventions that show an average of 10% improvement [31,32].

### Strengths and Limitations

The randomized controlled design with follow-up is rare in this field of research and can be considered a strength in this study [33]. However, dropout rate in our study, although comparable with a previous study among students [16] and yet still higher than in a study involving professionals [22], was a significant limitation and may represent diminished student satisfaction regarding e-learning [34]. In addition, differential dropout occurred across conditions, possibly because of motivational reasons. Furthermore, the evaluations of guideline adherence, confidence, and knowledge were self-reported, which are subject to the effects of social desirability and demand characteristics, although fairly reliable [35,36]. To get a more objective outcome, one could use role-plays in which a participant interacts with a patient with suicidal tendencies; however, this is much more time consuming. Although this study showed the effectiveness of e-learning in a waitlist control design, its relative effectiveness to alternative research-validated suicide prevention training programs [37] remains unclear and worth studying, especially considering the potential cost- and resource-efficiency of e-learning. Finally, it remains important to question how well the results obtained in a volunteer student sample generalize to working professionals required to take the course. However, as mentioned previously, similar results were found for mental health professionals in our previous study [23], indicating promising generalizability for the workforce.

### Implications and Future Research

E-learning modules, as the one used in this study, may be a beneficial, accessible way to provide training in suicide prevention skills to both students and professionals [38]. We propose to use e-learning modules in training suicide prevention skills in a wide range of higher education disciplines, such as curricula in nursing, social work, psychology, and psychiatry, to improve the knowledge and skills of clinicians-in-training regarding suicide prevention on a greater scale and consequently improve the care of patients with suicidal thoughts. To evaluate the transfer of knowledge and skills, and competencies of the clinicians (in training) to deal with clients’ suicidal behavior and subsequently reduce suicidality rates, future studies should include (objective) measures for clinician behavior. In addition, careful logging of content exposure and participant adherence to the program is advised for future studies to evaluate the extent to which the effects are attributable to the actual intervention as well as to evaluate the acceptability and feasibility of the intervention for future implementation. Because evidence of the cost-effectiveness of Web-based education in students is still lacking [14], future studies on the benefits of e-learning should consider (the reduction in) costs of personnel, education, and implementation to evaluate the cost-effectiveness. This study, with a randomized design and a feasible sample size of undergraduate students, provides first evidence of the positive effects of acquainting students on a large scale with the suicide prevention guidelines by means of an e-learning module. An e-learning module on the prevention of suicide could be an effective first step toward the improvement of clinical skill knowledge [39]. The learning outcomes of a stand-alone module were found to be similar to those of a training that combined e-learning with a face-to-face training, with the advantages of flexibility and low costs [15,30]. Furthermore, the students found the effects of the module to be reasonably positive; they believed that it prepared them well for their internship or job, and they were willing to recommend the module to colleagues and fellow students.

## Abbreviations

e-learning: electronic learning
PITSTOP: Professionals in Training to STOP
T0: baseline
T1: 1 month after baseline
T2: 3 months after baseline
VU: Vrije Universiteit
